# Correction: Iodine-mediated synthesis of indolyl-1,3,4-thiadiazole amine derivatives and their DFT analysis

**DOI:** 10.1039/d5ra90063h

**Published:** 2025-05-23

**Authors:** Vartika Vaishya, Manish K. Mehra, Sarvesh Kumar Pandey, Meenakshi Pilania

**Affiliations:** a Department of Chemistry, School of Physical and Biological Sciences, Manipal University Jaipur VPO-Dehmi-Kalan, Off Jaipur-Ajmer Express Way Jaipur Rajasthan 303007 India meenakshi.pilania@jaipur.manipal.edu meenakshi.pilania1987@gmail.com; b Department of Chemistry, Gachon University 1342 Seongnamdaero Seongnam Gyeonggi 13120 Republic of Korea; c Department of Chemistry, Maulana Azad National Institute of Technology Bhopal Bhopal Madhya Pradesh 462003 India

## Abstract

Correction for ‘Iodine-mediated synthesis of indolyl-1,3,4-thiadiazole amine derivatives and their DFT analysis’ by Vartika Vaishya *et al.*, *RSC Adv.*, 2025, **15**, 15108–15115, https://doi.org/10.1039/D5RA02026C.

The authors regret that the name of one of the authors (Sarvesh Kumar Pandey) was shown incorrectly in the original article. The corrected author list is as shown above.

The Royal Society of Chemistry apologises for these errors and any consequent inconvenience to authors and readers.

